# Phylloremediation of Air Pollutants: Exploiting the Potential of Plant Leaves and Leaf-Associated Microbes

**DOI:** 10.3389/fpls.2017.01318

**Published:** 2017-07-28

**Authors:** Xiangying Wei, Shiheng Lyu, Ying Yu, Zonghua Wang, Hong Liu, Dongming Pan, Jianjun Chen

**Affiliations:** ^1^Fujian Univeristy Key Laboratory of Plant-Microbe Interaction, College of Life Science, Fujian Agriculture and Forestry University Fuzhou, China; ^2^Department of Environmental Horticulture and Mid-Florida Research and Education Center, Institute of Food and Agricultural Sciences, University of Florida Apopka, FL, United States; ^3^College of Horticulture, Fujian Agriculture and Forestry University Fuzhou, China; ^4^College of Resource and Environmental Science, Fujian Agriculture and Forestry University Fuzhou, China

**Keywords:** air pollution, nitrogen dioxides, ozone, particulate matter, phylloremediation, phyllosphere, sulfur dioxide, volatile organic compounds

## Abstract

Air pollution is air contaminated by anthropogenic or naturally occurring substances in high concentrations for a prolonged time, resulting in adverse effects on human comfort and health as well as on ecosystems. Major air pollutants include particulate matters (PMs), ground-level ozone (O_3_), sulfur dioxide (SO_2_), nitrogen dioxides (NO_2_), and volatile organic compounds (VOCs). During the last three decades, air has become increasingly polluted in countries like China and India due to rapid economic growth accompanied by increased energy consumption. Various policies, regulations, and technologies have been brought together for remediation of air pollution, but the air still remains polluted. In this review, we direct attention to bioremediation of air pollutants by exploiting the potentials of plant leaves and leaf-associated microbes. The aerial surfaces of plants, particularly leaves, are estimated to sum up to 4 × 10^8^ km^2^ on the earth and are also home for up to 10^26^ bacterial cells. Plant leaves are able to adsorb or absorb air pollutants, and habituated microbes on leaf surface and in leaves (endophytes) are reported to be able to biodegrade or transform pollutants into less or nontoxic molecules, but their potentials for air remediation has been largely unexplored. With advances in omics technologies, molecular mechanisms underlying plant leaves and leaf associated microbes in reduction of air pollutants will be deeply examined, which will provide theoretical bases for developing leaf-based remediation technologies or phylloremediation for mitigating pollutants in the air.

## Introduction

Air pollution is referred to as the presence of harmful or poisonous substances in the earth's atmosphere, which cause adverse effects on human health and on the ecosystem. Major air pollutants include particulate matters (PMs), nitrogen oxides (NO_2_), sulfur dioxide (SO_2_), ground-level ozone (O_3_), and volatile organic compounds (VOCs) (Archibald et al., 2017). Various effects of some common air pollutants on human comfort and health are presented in Table 1, ranging from respiratory illness, cardiovascular disease to bladder and lung cancer (Kampa and Castanas, 2008).

**Table 1:** Major air pollutants and their effects on human comfort and health.

**Pollutants**	**Adverse effects on human health**	**References**
PMs^a^	Heart and lung cancer, risk of bladder cancer, nervous systems and respiratory illness	Anderson et al., 2012; Kumar et al., 2013; WHO, 2013; Kelly and Fussell, 2015
O_3_	Breathing problems, asthma, reduction of lung function, and lung diseases	Amann, 2008
NO_2_	Asthmatic bronchitis, reduced lung function growth, respiratory infections, and airway obstruction	Lambert et al., 1993; Lambert, 1996; Eberlein-Konig et al., 1998
SO_2_	Eye irritation, infections of the respiratory tract, coughing, mucus secretion, asthma and chronic bronchitis, and cardiac disease	Qin et al., 1993
CO	Neuropsychological impairment, headache, fatigue, dizziness, and nausea, fetal damage, myocardial ischemia	Dahms et al., 1993
PAHs^b^	Lung cancer	Fugas and Sega, 1995
Volatile organic compounds	Asthma, nocturnal breathlessness, sensitization reactions, respiratory tract, mucous membrane irritation, central nervous system symptoms, headache, drowsiness, fatigue, confusion, lethargy, and dizziness	Molhave, 1991; Wallace, 1991; Wieslander et al., 1996
Formaldehyde	Sneezing, coughing, minor eye irritation, irritant of the skin, respiratory tract, and nasopharyngeal cancer	Wieslander et al., 1996; Morgan, 1997; Eberlein-Konig et al., 1998
Radon	Lung cancer, acute myeloid and acute lymphoblastic leukemia	Steindorf et al., 1995
Tobacco smoke	Irritation of eye, nose, and throat, asthma, lung cancer, bronchitis, pneumonia, bronchiolitis, acute childhood lower respiratory tract illnesses, and tuberculosis	Lin et al., 2007
Asbestos	Skin irritation, lung cancer, mesothelioma, and asbestosis	McDonald, 1991

a *Particulate matters*.

b *Polycyclic aromatic hydrocarbons*.

The world has experienced unprecedented urban growth during the last three decades. Urban population is expected to increase at 2.3% per year in developing countries from 2000 to 2030 (Brockherhoff, 2000; United Nations, 2000, 2004; UNFPA, 2004). Urbanization is often associated with rapid economic growth. For example, China's urbanization grew from 17.92% in 1978 to 52.57% in 2012, and China's gross domestic products (GDPs) increased from 454.6 billion Chinese Yuan in 1980 to 51,894.2 billion Yuan in 2012 (Zhao and Wang, 2015). The increased economic growth has been accompanied with elevated energy consumption. China's energy consumption, primarily fossil fuels like coal, increased from 602.75 million tons in 1980 to 3,617.32 million tons in 2012 (Zhao and Wang, 2015). The increased combustion of fossil fuels with relatively low combustion efficiency along with weak emission control measures have resulted in drastic increases in air pollutants, such as PMs, SO_2_, NO_2_, O_3_, and VOCs. Per unit of GDPs in 2006, China emitted 6–33 times more pollutants than the United States (US). As a result, air quality has become a major focus of environmental policy in China. India experiences similar situations as China. Urbanization coupled with rapid economic development in India increased energy consumption and also air pollution in some megacities (Gurjar et al., 2016). For example, PM_10_ in Delhi was almost 10 times of the maximum PM_10_ limit at 198 μg m^−3^ in 2011 (Rizwan et al., 2013). Concentrations of major pollutants in the air of some selected cities are present in Table 2.

**Table 2:** Concentrations of some major air pollutants in the air of selected cities.

**City (country)**	**Year**	**PM_2.5_(μg m^−3^)**	**PM_10_(μg m^−3^)**	**O_3_(μg m^−3^)**	**NO_2_(μg m^−3^)**	**SO_2_(μg m^−3^)**	**CO (mg m^−3^)**	**References**
Ho Chi Minh City (Vietnam)	2007	−	74.00	40.00	18.90	30.30	–	Phung et al., 2016
Patras (Greece)	2011	−	42.10	84.20	47.10	–	0.90	Karagiannidis et al., 2014
Quetta (Pakistan)	2009	160.28	370.52	–	97.05	50.00	3.80	Ilyas et al., 2009
Beijing (China)	2015	78.50	104.82	–	50.49	16.86	1.25	Chen et al., 2016
Shanghai (China)	2015	55.54	75.64	–	45.23	18.40	0.86	Chen et al., 2016
Shenzhen (China)	2015	32.83	55.37	–	32.94	8.08	1.01	Chen et al., 2016
Guangzhou (China)	2015	44.38	65.91	–	45.46	14.67	0.99	Chen et al., 2016
Rome (Italy)	2015	19.50	27.60	42.30	48.70	1.00	0.60	Battista et al., 2016
Lucknow City (India)	2012	91.10	217.35	–	74.10	12.30	0.20	Lawrence and Fatima, 2014

The World Health Organization (WHO) air quality guidelines stated that the mean limits for annual exposure to PM_2.5_ (particle diameters at 2.5 μm or less) and PM_10_ (particle diameter at 10 μm or less) are 10 μg m^−3^ and 25 μg m^−3^, respectively; and the limits for 24-h exposure are 25 μg m^−3^ and 50 μg m^−3^, respectively. The limit for 8-h exposure to O_3_ is 100 μg m^−3^. Annual mean for NO_2_ is 40 μg m^−3^ or 200 μg m^−3^ for 1 h, and 24-h exposure to SO_2_ is 20 μg m^−3^ or 500 μg m^−3^ for 10 min (WHO, 2006). The results presented in Table 2 suggest that residents in some of the listed cities were exposed to air contamination far beyond the limits set by WHO. PMs have become the most pressing environmental problems in China and India. For example, during the first quarter of 2013, China experienced extremely severe and persistent haze pollution that directly affected about 1.3 million km^2^ and about 800 million people (Huang et al., 2014). Of which daily average concentrations of PM_2.5_ measured at 74 major cities exceeded the Chinese pollution standard of 75 μg m^−3^, which is approximately twice that of the US EPA (United States Environmental Protection Agency) standard of 35 μg m^−3^, for 69% of days in January, with a record-breaking daily concentration of 772 μg m^−3^ (Huang et al., 2014).

Recent studies from the International Agency for Research on Cancer showed that there were 223,000 deaths in 2010 due to air pollution-resultant lung cancer worldwide, and air pollution has become the most widespread environmental carcinogen (International Agency for Research on Cancer, 2013). The WHO reported that around 7 million people died of air pollution exposure directly or indirectly in 2012. This data was more than double previous estimates and confirmed that air pollution has become a substantial burden to human health and is the world's largest single environmental health risk (WHO, 2014). Additionally, air pollution also harms animals, plants, and ecological resources including water and soils (Vallero, 2014; Duan et al., 2017).

## Measures for reducing air pollution

To reduce air pollution, the first step is to eliminate or reduce anthropogenic-caused emissions. The second step is to remediate existing pollutants. Different strategies, policies, and models for air pollution abatement have been proposed or implemented (Macpherson et al., 2017). For example, the Chinese government has imposed restrictions on major pollution sources including vehicles, power plants, transport, and industry sectors (Liu et al., 2016) and promulgated the “Atmospheric Pollution Prevention and Control Action Plan” in September 2013, which was intended to reduce PM_2.5_ by 25% by 2017 relative to 2012 levels (Huang et al., 2014). Science-based technologies have been developed for control of air pollutants, such as diesel particulate filters (Tsai et al., 2011) and activated carbon filtering as adsorbent for xylene and NO_2_ (Guo et al., 2001). Catalytic oxidization and chemisorption methods have been used for indoor formaldehyde removal (Pei and Zhang, 2011; Wang et al., 2013). Photocatalysis as one of the most promising technologies has been used for eliminating VOCs (Huang et al., 2016).

Air pollutants can also be mitigated through biological means, commonly referred to as biological remediation or bioremediation. It is the use of organisms to assimilate, degrade or transform hazardous substances into less toxic or non toxic ones (Mueller et al., 1996). Plants have been used for remediation of pollutants from air, soils, and water, which has been termed as phytoremediation (Cunningham et al., 1995; Salt et al., 1995; Huang et al., 1997). Microbes such as bacteria and fungi are also capable of biodegrading or biotransforming pollutants into non toxic and less toxic substances, which is known as microbial biodegradation (Ward et al., 1980; Ma et al., 2016). Microbes as heterotrophs occur nearly everywhere, including plant roots and shoots. Both roots and shoots have been reported to be able to remediate air pollutants (Weyens et al., 2015; Gawronski et al., 2017), but little credit has been given to microbe activity.

Plant shoots or the above-ground organs of plants colonized by a variety of bacteria, yeasts, and fungi are known as phyllosphere (Last, 1955). However, most scientific work on phyllosphere microbiology has been focused on leaves (Lindow and Brandl, 2003). This review is intended to explore the potential of plant leaves and leaf-associated microbes in bioremediation of air pollutants, or simply known as phylloremediation. Phylloremediation was first coined by Sandhu et al. (2007), who demonstrated that surface-sterilized leaves took up phenol, and leaves with habiated microbes or a inoculated bacterium were able to biodegrade signficantly more phenol than leaves alone. Previous reports also documented that both plant leaves and leaf-associated microbes mitiagted air pollutants, such as azalea leaves and the leaf-associated *Pseudomonas putida* in reducing VOCs (De Kempeneer et al., 2004), leaves of yellow lupine plants along with endophytic *Burkholderia cepacia* for toluene reduction (Barac et al., 2004), and poplar leaves and the leaf-associated *Methylobacterium* sp. decreased xenobiotic compounds (Van Aken et al., 2004). Phyllo originated from Greek word of phullon, meaning leaf. Thus, phylloremediation should be defined as a natural process of bioremediation of air pollutants through leaves and leaf-associated microbes, not the microbes alone.

## Plant leaves and phyllosphere

**Figure 1:**
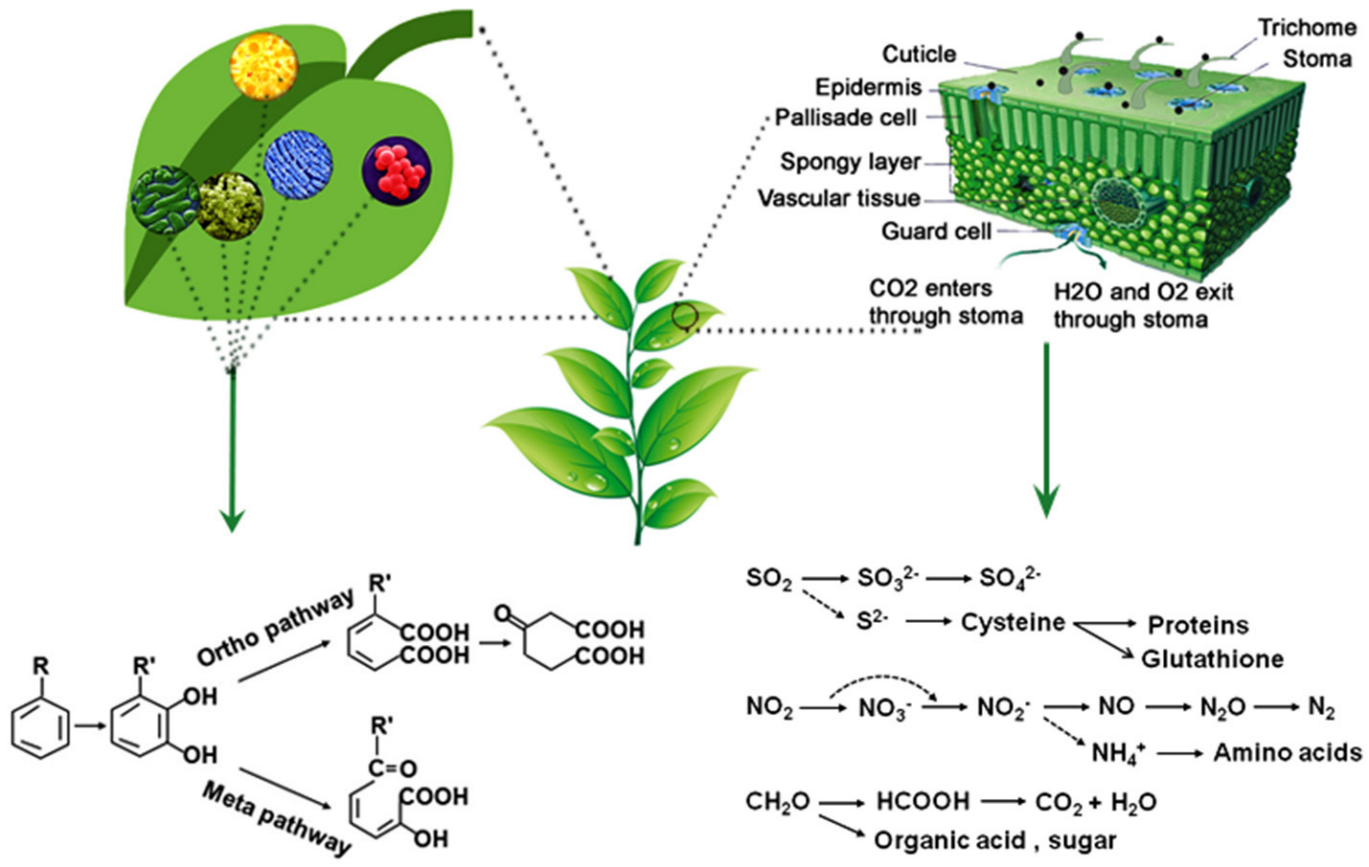
A schematic illustration of phyllosphere. The middle panel represent an aerial part of a plant. Right panel shows a magnified schematic cross section of a leaf where leaf surface and trichomes can retain particulate matter (PMs) and stomata adsorb or absorb PMs as well as how leaves can assimilate SO_2_, NO_2_, and CH_2_O (formaldehyde) to simple organic compounds, amino acids, or proteins. The left panel depict a magnified leaf surface with bacteria, which can biodegrade or transform volatile organic compounds to less toxic or nontoxic ones like benzene and its derivatives that can be degraded through Ortho pathway or Meta pathway.

Leaves are the primary photosynthetic organs with distinctive upper surface (adaxial) and lower surface (abaxial) (Figure 1). The upper surface has a layer (<0.1–10 μm) of waxy cover called cuticle (Kirkwood, 1999). Wax contents and compositions frequently differ among plant species. The primary function of cuticle is to prevent evaporation of water from leaf surfaces, and it is also the first barrier for the penetration of xenobiotics. The leaf surface is filled with trichomes, which are epidermal outgrowths in various forms. Trichomes play roles in mechanical defense because of their physical properties and also in biochemical defense due to the secretion of secondary metabolites (Tian et al., 2017). Epidermis cells are directly underneath the cuticle layer in which stomata often occur. Xylem and phloem are situated within the veins of leaves as the plant vascular system, which are connected from root tips to leaf edges. There is a layer of compactly arranged cells around the vein called bundle sheath regulating substance circle around the xylem and the phloem. Xylem transports water and nutrients from roots to shoots, and phloem transports assimilated products from source and sink tissues. Under the epidermis, there are mesophyll cells in two layers: column-like palisade cells and loosely packed spongy cells. The air spaces among the spongy cells promote gas exchange, and photosynthesis takes place in chloroplasts packed in the mesophyll cells. The underside of leaves also has a layer of epidermal cells where most stomata are located. There are two guard cells surround the stomata, and stomatal pore opening and closure is regulated by changes in the turgor pressure of the guard cells. Stomata regulate the flow of gases in and out of leaves and also able to adsorb or absorb other chemicals.

Leaves also play pivotal roles in supporting phyllosphere microbes (Bringel and Couee, 2015). The phyllosphere is estimated to have area up to 4 × 10^8^ km^2^ on the earth and is the home for up to 10^26^ bacterial cells (Kembel et al., 2014). Phyllosphere bacterial communities are generally dominated by Proteobacteria, such as *Methylobacterium* and *Sphingomonas*. *Beijerinckia, Azotobacter, Klebsiella*, and Cyanobacteria like *Nostoc, Scytonema*, and *Stigonema* also reside in the phyllosphere (Vacher et al., 2016). Population of γ-Proteobacteria such as *Pseudomonas* could be high as well (Delmotte et al., 2009; Fierer et al., 2011; Bodenhausen et al., 2013; Kembel et al., 2014). Dominant fungi in the phyllosphere include Ascomycota, of which the most common genera are *Aureobasidium, Cladosporium*, and *Taphrina* (Coince et al., 2013; Kembel and Mueller 2014). Basidiomycetous yeasts belonging to the genera *Cryptoccoccus* and *Sporobolomyces* are also abundant in phyllosphere (Cordier et al., 2012; Ottesen et al., 2013). The microbes can be epiphytic by living on the surface of plant organs and/or endophytic occurring within plant tissues without causing apparent disease.

Plant species significantly influence the composition of a phyllosphere community (Whipps et al., 2008). In a study of 56 different tree species, Redford et al. (2010) reported that different species harbor distinct microbial communities in phyllosphere. This principle was also confirmed for trees in temperate and tropical climates and for Mediterranean perennials (Lambais et al., 2006; Kim et al., 2012; Vokou et al., 2012; Kembel et al., 2014; Laforest-Lapointe et al., 2016). Using high-throughput sequencing technology, Kembel and Mueller (2014) studied fungal communities on leaves of 51 tree species in a lowland tropical rainforest in Panama and reported that fungal communities on leaves were dominated by the phyla Ascomycota, which accounted for 79% of all sequences, followed by Basidiomycota (11%) and Chytridiomycota (5%). More than half of the variation in fungal community composition could be explained by plant species differences. Leaf chemistry and morphology as well as plant growth status and mortality were closely related to fungal community structure (Kembel and Mueller, 2014). These results may suggest that different tree species host different fungal communities. Additionally, microbial compositions within plant species may differ due to geographic locations (Finkel et al., 2012; Qvit-Raz et al., 2012; Rastogi et al., 2012). The differences could be caused by climatic variation (Finkel et al., 2011) or due to the limited dispersal of the colonizing taxa (Finkel et al., 2012; Qvit-Raz et al., 2012). Furthermore, phyllosphere microbial community may differ between urban and non-urban locations (Jumpponen and Jones, 2010) and also differ by seasons (Redford and Fierer, 2009).

## Roles of leaves and phyllosphere microbes in air remediation

The close association between plant species and specific microbial communities in the phyllosphere suggests their adaptation and coevolutionary relationships. Recent studies show that leaf bacterial diversity mediates plant diversity and ecosystem function relationships (Laforest-Lapointe et al., 2017). We hypothesize that a long-lasting exposure of leaves and leaf-associated microbes to air pollutants could result in plants or microbes individually or coordinately developing mechansims for adapting to the polluted substances. Such mechanisms may include leaf adsorption or absorption and pollutant assimilation as well as microbial biodegradation, transformation or metabolic assimilation of the substances. The coordination between leaves and micriobes could be synergistic or antagonistic. Table 3 presents plant-supported microbes that are able to biodegrade or biotransform air pollutants, primarily organic compounds. However, information regarding phyllospere microbes in remediation of PMs, SO_2_, NO_2_, and O_3_ is scarce, suggesting relatively limited research has been devoted to microbial roles. Thus, the current knowledge on phylloremediation of PM, SO_2_, NO_2_, and O_3_ is mostly come from plants.

**Table 3:** Plant-supported microbes that are able to biodegrade or biotransform air pollutants.

**Plants**	**Microbes**	**Pollutant**	**References**
*Acorus calamus* var. *angustatus*	*Methylobacterium* sp.	Methanol	Iguchi et al., 2012
*Amaranthus cruentus*	*Alcaligenes feacalis* and *Alcaligenes* sp.11SO	Naphthalene and Phenanthrene	Ljs, 2016
*Annona muricana*	*Pseudomonas* sp, *Alcaligenes* sp, and *Microcccus roseus*	Hydrocarbon	Ilori et al., 2006
*Arabidopsis thaliana*	*Hyphomicrobium* sp,	Chloromethane	Nadalig et al., 2011
*Arabidopsis thaliana*	*Achromobacter xylosoxidans* F3B	Phenolic pollutants	Ho et al., 2012
*Arachis hypogaea*	*Arthrobacter nitroguajacolicus, Achromobacter xylosoxidans, Basea thiooxidans, Microbacterium natoriense, Kocuria rosea, Dyadobacter fermentans*, and *Klebsiella pneumoniae*	n-alkanes	Al-Awadhi et al., 2012
*Azalea indica*	*Pseudomonas putida* TVA8	Toluene	De Kempeneer et al., 2004
*Boerhavia diffusa*	*Rhodococcus corynebacteriodes* and *Exiguobacterium arabatum*	n-alkanes	Al-Awadhi et al., 2012
*Bougainvillea buttiana*	*Enterobacter cloacae* LSRC11, *Staphylococcus* sp. A1 and *Pseudomonas aeruginos*	Xylene	Sangthong et al., 2016
*Calystegia soldanella*	*Hyphomicrobium* sp.	Methanol	Iguchi et al., 2012
*Chenopodium album*	*Burkholderia fungorum*	n-alkanes	Al-Awadhi et al., 2012
*Chenopodium murale*	*Gordonia polyisoprenivorans*	n-alkanes	Al-Awadhi et al., 2012
*Chenopodium murale*	*Flavobacterium* sp., *Halomonas* sp., and *Arthrobacter* sp.	Volatile hydrocarbons	Ali et al., 2015
*Chrysopogon zizanioides*	*Achromobacter xylosoxidans* F3B	BTEX compounds (benzene, toluene, ethylbenzene, and xylene)	Ho et al., 2013
*Clitoria ternatea*	*Bacillus cereus*	Formaldehyde	Khaksar et al., 2016b
*Conocarpus lancifolius*	*Flavobacterium* sp., *Halomonas* sp., and *Arthrobacter* sp.	Volatile hydrocarbons	Ali et al., 2015
*Cucumis sativus*	*Arthrobacter ureafaciens, Arthrobacter aurescens*, and *Microbacterium natoriense*	n-alkanes	Al-Awadhi et al., 2012
*Cynodon* spp.	*Rhodococcus* sp. and *Pseudomonas* sp.	Volatile oil hydrocarbons crude oil, n-hexadecane, or phenanthrene	Sorkhoh et al., 2011
*Eichhornia crassipes*	*Methylophilus* sp.	Methanol	Iguchi et al., 2012
*Ervatamia divaricata*	*Alcaligenes feacalis* and *Alcaligenes* sp.11SO	Naphthalene and Phenanthrene	Ljs, 2016
*Fraxinus excelsior*		Trichloroethylene, Toluene	Weyens et al., 2009a
*Fraxinus pennsyhanica*	*Acinetobacter* sp., *Alcaligenes* sp., and *Rhodococcus* sp.	Phenol	Sandhu et al., 2009
*Gazania rigens*	*Methylobacterium populi, Gordonia lacunae, Dietzia maris, Microbacterium oleivorans*, and *Pseudomonas stutzeri*	n-alkanes	Al-Awadhi et al., 2012
*Gazania rigens*	*Flavobacterium* sp., *Halomonas* sp., and *Arthrobacter* sp.	Volatile hydrocarbons	Ali et al., 2015
*Gossipium hirsutum* ‘MCU12’	*Methylobacterium gossipiicola* sp. nov.	Methanol and dichloromethane	Madhaiyan et al., 2012)
*Hibiscus rosa-sinensis*	*Alcaligenes feacalis* and *Alcaligenes* sp.11SO	Naphthalene and Phenanthrene	Ljs, 2016
*Hedera* spp.	*Hymenobacter* sp. and *Sphingomonadaceae* sp.	PMs	Smets et al., 2016
*Ixora chinensis*	*Alcaligenes feacalis* and *Alcaligenes* sp.11SO	Naphthalene and Phenanthrene	Ljs, 2016
*Ixora* spp.	*Acinetobacter* sp. and *Pseudomonas* sp. *Pseudoxanthomonas* sp., and *Mycobacterium* sp.	Polycyclic aromatic hydrocarbons	Yutthammo et al., 2010
*Ixora* spp.	*Pseudomonas* sp., *Microbacterium* sp., *Rhizobium* sp., and *Deinococcus* sp.	Phenanthrene	Waight et al., 2007
*Keteleeria davidiana*	*Methylobacterium* sp. and *Methylophilus* sp.	Methanol	Iguchi et al., 2012
*Lolium multiflorum Lam*.	*Pseudomonas* sp. Ph6-gfp	Phenanthrene	Sun et al., 2014
*Lolium multiflorum* var. Taurus	*Pseudomonas* sp. ITSI10, ITRI15, ITRH76, and BTRH79	Hydrocarbon	Yousaf et al., 2010
*Lolium perenne*	*Pseudomonas* sp.	Petroleum hydrocarbons	Kukla et al., 2014
*Lotus corniculatus* var. Leo	*Pseudomonas* sp. ITSI10, ITRI15, ITRH76, and BTRH79	Hydrocarbon	Yousaf et al., 2010
*Magnifera indica*	*Pseudomonas* sp., *Alcaligenes* sp., and *Microcccus roseus*	Hydrocarbon	Ilori et al., 2006
*Malus pumila*	*Arthrobacter* sp.	4-chlorophenol	Scheublin and Leveau, 2013
*Mattiola incana*	*Acinetobacter calcoacelicus, Pseudomonas putida, Planomicrobium glaciei,Arthrobacter agilis, Kucuria turfanensis, Pseudomonas geniculate, Chyseobacterium taeanense, Flavobacterium ahuensis*, and *Microbacterium oxydans*	n-alkanes	Al-Awadhi et al., 2012
*Mesembryanthemum nodifloru*	*Nesterenkonia jeotgali, Nesterkonia lacusekhoensis*, and *Agrococcus terreus*	n-alkanes	Al-Awadhi et al., 2012
*Oryza sativa*	*Methylobacterium* sp.	Methanol	Knief et al., 2012
*Phaseolus vulgaris*	*Microbacterium* sp. and *Citrobacter freundii*	Crude oil, phenanthrene and n-octadecane	Ali et al., 2012
*Phaseolus vulgaris*	*Arthrobacter chlorophenolicus*	4-chlorophenol, hydroquinone	Scheublin et al., 2014
*Phaseolus vulgaris*	*Pseudomonas* sp. CF600	Phenol	Sandhu et al., 2007
*Phragmites australis*	*Hyphomicrobium* sp.	Methanol	Iguchi et al., 2012
*Picea abies*		Trichloroacetic acid	Forczek et al., 2004
*Pisum sativum*	*Pseudomonas putida* VM1441(pNAH7)	Polyaromatic hydrocarbons (PAHs)	Germaine et al., 2009
*Pisum sativum*	*Microbacterium* sp. and *Rhodococcus* sp.	Crude oil, phenanthrene and n-octadecane	Ali et al., 2012
*Pisum sativum*	*Agromyces fucosus, Agrococcus jenensis, Paenibacillus polymixa, Bacillus cereus, Bacillus megaterium, Brevibacillus brevis, Bacillus, Neolsonii, Bacillus subtilis, Arthrobacter ramosus, Microbacterium imperial, Bacillus endophyticus*, and *Cellulosimicrobium cellulans*	n-alkanes	Al-Awadhi et al., 2012
*Poaceae* spp.	*Hyphomicrobium* sp.	Methanol	Iguchi et al., 2012
*Populus deltoides*	*Pseudomonas putida* W619-TCE	Trichloroethylene (TEC)	Weyens et al., 2009b
*Populus deltoids* × *nigra*	*Methylobacterium* sp.	2,4,6-trinitrotoluene, hexahydro-1,3,5-trinitro-1,3,5-triazine, and octahydro-1,3,5,7-tetranitro-1,3,5-tetrazocine	Van Aken et al., 2004
*Populus trichocarpa deltoides*	*Burkholderia cepacia* VM1468	Toluene	Taghavi et al., 2005
*Pyrus calleryansa*	*Methylobacterium* sp. and *Methylophilus* sp.	Methanol	Iguchi et al., 2012
*Quercus phillyraeoides*	*Methylobacterium* sp. and *Methylophilus* sp.	Methanol	Iguchi et al., 2012
*Quercus robu*		Trichloroethylene, toluene	Weyens et al., 2009a
*Salix discolor* clone S-365	*Pseudomonas putida* PD1	Polycyclic aromatic hydrocarbons (PAHs)	Khan et al., 2014
*Salix purpurea clone* 94006	*Pseudomonas putida* PD1	Polycyclic aromatic hydrocarbons (PAHs)	Khan et al., 2014
*Salsola baryosma*	*Halomonas marisflava* and *Salinococcus hispanicus*	n-alkanes	Al-Awadhi et al., 2012
*Sonchus oleraceus*	*Flavobacterium* sp., *Halomonas* sp., and *Arthrobacter* sp.	Volatile hydrocarbons	Ali et al., 2015
*Tecoma stans*	*Afipia genosp* and *Microbacterium hydrocarbonoxydans* and *Bacillus subtilis*	n-alkanes	Al-Awadhi et al., 2012
*Tecoma stans*	*Flavobacterium* sp., *Halomonas* sp. and *Arthrobacter* sp.	Volatile hydrocarbons	Ali et al., 2015
*Vicia faba*	*Flavobacterium* sp., *Halomonas* sp. and *Arthrobacter* sp.	Volatile hydrocarbons	Ali et al., 2015
*Vicia faba*	*Rhodococcus* sp. and *Pseudomonas* sp.	Volatile oil hydrocarbons crude oil, n-hexadecane, or phenanthrene	Sorkhoh et al., 2011
*Vigna unguiculata*	*Microbacterium arabinogalactanolyticum* and *Pseudomonas oryzihabtans*	n-alkanes	Al-Awadhi et al., 2012
*Viola* x *wittrockiana*	*Methylobacterium* sp. and *Methylophilus* sp.	Methanol	Iguchi et al., 2012
*W. religiosa*	*Acinetobacter* sp., *Pseudomonas* sp., *Pseudoxanthomonas* sp. and *Mycobacterium* sp.	Polycyclic aromatic hydrocarbons	Yutthammo et al., 2010
*Zamioculcas zamiifolia*	*Bacillus cereus*	Formaldehyde	Khaksar et al., 2016a
*Zamioculcas zamiifolia*	*Pseudomonas aeruginosa* and *Bacillus cereus*	Ethylbenzene	Toabaita et al., 2016
*Zea mays*	*Pseudomonas* sp. CF600	Phenol	Sandhu et al., 2007

### Remediation of PMs

As mentioned above, PMs have become the most dangerous pollutants in some countries. Chemical species of PMs, derived from the available data over China included ${\text{SO}}_{4}^{2-}$, ${\text{NO}}_{3}^{-}$, ${\text{NH}}_{4}^{+}$, organic carbon, and elemental carbon, which were in a range of 2.2–60.9, 0.1–35.6, 0.1–29.8, 1.5–102.3, 0.2–37.0 μg cm^−3^ in PM_2.5_, and 1.6–104.6, 0.5–46.6, 0.2–31.0, 1.7–98.7, and 0.3–26.8 μg cm^−3^ in PM_10_, respectively (Zhou et al., 2016). PM_2.5_ is the major component of PM_10_, accounting for 65%. PMs are also composed of microorganisms. In a study of PMs in Jeddah, Saudi Arabia (Alghamdi et al., 2014), the average concentrations of PM_10_ and PM_2.5_ were 159.9 and 60 μg cm^−3^, respectively and the concentrations of O_3_, SO_2_, and NO_2_ averaged 35.73, 38.1, and 52.5 μg cm^−3^, respectively. Microbial loads were higher in PM_10_ than PM_2.5_. *Aspergillus fumigatus* and *Aspergillus niger* were the common fungal species associated with PMs. Microbes were also found in PMs in Austria (Haas et al., 2013), including fungi from genera *Aspergillus, Cladosporium*, and *Penicillium* and aerobic mesophilic bacteria. Using metagenomic methods, Cao et al. (2014) identified 1,315 distinct bacterial and archaeal species from 14 PM samples collected from Beijing, China. The most abundant phyla were Actinobacteria, Proteobacteria, Chloroflexi, Firmicutes, Bacteroidetes, and Euryarchaeota. Among them, an unclassified bacterium in the nitrogen fixing, filamentous bacteria genus Frankia was the most abundant, and the most abundant classified bacterial species appeared to be *Geodermatophilus obscures*. The abundance of airborne bacteria was reported to be in a range from 10^4^ to 10^6^ cells m^−3^ depending on environmental conditions (Bowers et al., 2011), and materials of biological origin might account for up to 25% of the atmospheric aerosol (Jaenicke, 2005). Ammonia oxidizing archaea (AOA), ammonia oxidizing bacteria (AOB), and complete ammonia oxidizers (Comammox) were identified in PM_2.5_ collected from the Beijing-Tianjin-Heibei megalopolis, China (Gao et al., 2016). Of which *Nitrosopumilus* subcluster 5.2 was the most dominant AOA, *Nitrosospira multiformis* and *Nitrosomonas aestuarii* were the most dominant AOB, and the presence of Comammox was revealed by the occurrence of *Candidatus* Nitrospira inopinata. The mean cell numbers of AOA, AOB, and *Ca*. N. inopinata were 2.82 × 10^4^, 4.65 × 10^3^, and 1.15 × 10^3^ cell m^−3^, respectively. The average maximum nitrification rate of PM_2.5_ was 0.14 μg (NH4^+^-N) [m^3^ air h]^−1^ (Gao et al., 2016). AOA might account for most of the ammonia oxidation, followed by Comammox, while AOB were responsible for a small part of ammonia oxidation. The assay of nitrification activity was performed in laboratory conditions (Gao et al., 2016). However, the nitrification potential of such bacteria in PMs after being deposited on leaf surfaces is unknown. We hypothesize that the nitrification process could be more active once such PM-containing bacteria settled on leaves. Further investigation on nitrification of PM-associated bacteria in the phyllosphere could provide insight into how the phyllosphere could potentially act as manufactories in the nitrification of ammonia.

The current literature regarding phylloremediation of PMs has been primarily focused on plant leaves. Plant canopy is a sink for PMs. This is due to the fact that leaves are in the air and they span more than 4 × 10^8^ km^2^ on a global scale, which is about 78.4% of the total surface area of the earth; leaves thus physically act as a natural carrier for PMs. Leaves differ greatly in surface structure and metabolic secreted substances as well as microbial composition. The amount of surface waxes and compositions show different capacity to retain and embrace PMs. Sbø et al. (2012) studied leaves of 22 trees and 25 shrubs in accumulation of PMs in Norway and Poland and found that PM accumulation differed by 10 and 15 folds depending on plant species in the two locations and also positive correlations occurred among PM accumulation, leaf wax contents, and leaf hair density. Thirteen woody species were examined by Popek et al. (2013) during a 3-year period, and total amount of PMs captured by leaves ranged from 7.5 mg cm^−2^ by *Catalpa bignonioides* to 32 mg cm^−2^ by *Syringa meyeri*. Leaf wax contents were significantly correlated with the amount of PMs on leaves. Among the PMs captured, 60% was washable by water, and 40% could be washed by chloroform only, suggesting that the PMs were embraced in waxes. Using two photon excitation microscopy (TPEM), Terzaghi et al. (2013) investigated leaves of stone pine (*Pinus pinea*), cornel (*Cornus mas*), and maple (*Acer pseudoplatanus*) in capture and encapsulation of PMs. The authors found that particles ranging from 0.2 to 70.4 μm were visualized on leaves, of which PM_2.6_ was the dominant size across plant species. Particle less than 10.6 μm were encapsulated in the cuticle. Plant species differed in particle retention and encapsulation, which were attributed to leaf characteristics, cuticle chemical composition and structure.

Leaf physical characteristics such as leaf shape, hairs or trichomes, and stomata significantly affect PM accumulation. Needle leaves were reported to accumulate more PM_2.5_ than broad leaves (Terzaghi et al., 2013; Chen et al., 2017). The effectiveness was attributed to the higher capture efficiency and higher Stoke's numbers of needles compared to those of broad leaves (Beckett et al., 2000). Additionally, small individual leaf area and abundant wax layer also contribute to the effectiveness (Chen et al., 2017). Leaf trichomes have been shown to increase PM_2.5_ accumulation. The trichome density was positively correlated with amount of PM_2.5_ accumulated on leaves, and plant species with abundant hairs, such as *Catalpa speciosa, Broussonetia papyrifera*, and *Ulmus pumila* were able to retain more PM_2.5_ than those with fewer hairs (Chen et al., 2017). The adaxial surface of leaves accumulated more PMs than the abaxial leaf surface (Baldacchini et al., 2017), which is probably due to the fact that the abaxial surface in general has few trichomes and less rough surface. Stomata may play some roles in accumulation of PMs. The length of stomata ranges from 10 to 80 μm and densities varies from 5 to 1,000 mm^−2^ depending on plant species and environmental conditions (Hetherington and Woodward, 2003). Stomatal pore areas range from 46 to 125 μm^2^ (Peschel et al., 2003; Dow et al., 2014), thus stomata could retain or adsorb either PM_2.5_ or PM_10_. A study of PM deposition on leaves of five evergreen species in Beijing, China showed that PM diameter up to 2 μm was in the stomatal cavity (Song et al., 2015). Rai (2016) studied the effects of PMs on 12 common roadside plant species and found that stomatal sizes were reduced due to air dust deposition, but plant growth was not affected, suggesting the potential of plants in adsorbing air pollutants.

Growing evidence has suggested that plant leaves are able to capture PMs and act as biofilters. On average, the upper leaf surface of 11 plant species intercepted 1,531 particles per mm^−2^ (Wang et al., 2006). Needles of *Pinus sylvestris* accumulated 18,000 mineral particles per mm^2^ (Teper, 2009). Upper leaves of *Hedera helix* captured about 17,000 particles per mm^2^ (Ottele et al., 2010). Trees removed 1,261 tons of air pollutants in Beijing, of which 772 tons were PM_10_(Yang et al., 2005). In New Zealand, urban trees removed 1,320 tons of particular matter annually due to the existence of woodlands in Auckland (Cavanagh and Clemons, 2006). Nowak et al. (2014) showed trees within cities removed fine particles from the atmosphere and consequently improved air quality and human health. Tree effects on PM_2.5_ concentrations and human health are modeled for 10 U.S. cities. The total amount of PM_2.5_ removed by trees varied from 4.7 tons in Syracuse to 64.5 tons in Atlanta in the U.S annually. All the reported removal of PMs is attributed to plant leaves. It is unknown at this time if phyllosphere microbes could break down the PMs on leaves and if mineral elements released from the broken PMs could become plant nutrients. Considering the fact that the microbes can biodegrade a wide range of substances including petroleum, we hypothesize that some microbes should be able to break down PM. Future research in this regard will be conducted, and identified microbes could be used for PM reduction.

### Remediation of SO_2_

Sulfur dioxide (SO_2_) was among the first air pollutants identified to harm human health and ecosystems. The combustion of fossil fuels has substantially increased SO_2_ in the air. China has contributed to about one-fourth of global SO_2_ emission since 1990 (Zhang et al., 2013). The emission of SO_2_ from Guangdong province totaled 1,177 Gg in 2007, of which 97% was emitted by power plants and industries (Lu et al., 2010). SO_2_ can be oxidized photochemically or catalytically to sulfur trioxide (SO_3_) and sulfate (${\text{SO}}_{4}^{2-}$) in the air (Bufalini, 1971). With the presence of water, SO_3_ is converted rapidly to sulfuric acid (H_2_SO_4_), which is commonly known as acid rain. While in sulfur assimilation, ${\text{SO}}_{4}^{2-}$ is reduced to organic sulfhydryl groups (R-SH) by sulfate-reducing bacteria, fungi, and plants. Sulfur oxidizing bacteria such as *Beggiatoa* and *Paracoccus* are able to oxidize reduced sulfur compounds like H_2_S to inorganic sulfur, and thiosulfate to form sulfuric acid (Pokoma and Zabranska, 2015). Sulfate reducing bacteria like *Archaeoglobus* and *Desulfotomaculum* can convert sulfur compounds to hydrogen sulfide (H_2_S). Oxidation of H_2_S produces elemental sulfur (S°), which is completed by the photosynthetic green and purple sulfur bacteria and some chemolithothrophs. Further oxidation of elemental sulfur produces sulfate. Sulfate is assimilated through the sulfate activation pathway, which is consisted of three reactions: the synthesis of adenosine 5′-phosphorylation of (APS), the hydrolysis of GTp, and the 3′-phosphorylation of APS to produce 3′-phosphoadenosine 5′-phosphosulfate (PAPS) (Sun et al., 2005). In *Mycobacterium tuberculosis*, the entire sulfate activation pathway is organized into a single complex (Sun et al., 2005). Additionally, sulfate reducing bacteria have been shown to use hydrocarbons in pure cultures, which can be used for bioremediation of benzene, toluene, ethylbenzene, and xylene in contaminated soils (Muyzer and Stams, 2008). Such bacteria may also colonize leaf surfaces and could be used for remediation of air pollutants.

Plant leaves absorb SO_2_ via stomata. At apoplastic pH, it is hydrated and oxidized successively to sulfite and sulfate, both of which can inhibit photosynthesis and energy metabolism if they accumulate to a high concentration. Such inhibition can cause SO_2_ toxicity. Symptoms include interveinal chlorosis and necrosis in broad-leaved species, and chlorotic spots and brown tips in pine conifers (Rennenberg, 1984). Until the 1970s, SO_2_ was considered to be a key contributor of acid rain causing forest dieback (Bloem et al., 2015). Interestingly, when the Clean Air Acts came into action in the 1980s, the reduction in atmosphere SO_2_ resulted in sulfur (S) deficiency in crops, particularly *Brassica* species. The S deficiency was responsible for the increased incidence of disease caused by *Pyrenopeziza brassicae* (Bloem et al., 2015). The explanation is that plants could become injured in a SO_2_ concentration range from 131 to 1,310 μg m^−3^; plants, however, can rapidly assimilate SO_2_ and H_2_S into reduced sulfur pools such as cysteine and sulfates as illustrated in Figure 1. A recent transcriptome analysis of *Arabidopsis* responses to SO_2_ showed that plant adaptation to SO_2_ evokes a comprehensive reprogramming of metabolic pathways including NO and reactive oxygen species (ROS) signaling molecules, and also plant defense response pathways (Zhao and Yi, 2014). The importance of this study revealed that plant responses to SO_2_ stress is at the transcription level with initial activation of cross tolerance and followed by sulfur assimilation pathways. Cysteine metabolism in particular is associated with the network of plant stress responses, thus improving plant growth in soils where sulfur supply is limited (Bloem et al., 2015). It has been shown that an atmospheric level of 79 ng m^−3^ SO_2_ could contribute to 10–40% of leaf sulfur assimilation (De Kok et al., 2007; Zhao et al., 2008). Elevated SO_2_ concentrations around natural CO_2_ springs have been documented to enhance accumulation of sulfur metabolites and proteins in surrounding vegetation (Rennenberg, 1984). Therefore, plants can be selected for growing in SO_2_ polluted environments (Chung et al., 2010). In 2000, about 42.62 Mg of SO_2_ was removed from the atmosphere by urban trees in Guangzhou, China (Zhang et al., 2013). Additionally, S metabolism can be genetically engineered for improving plant resistance to SO_2_. Transgenic tobacco plants overexpressing cysteine synthase or serine acetyltransferase gene were highly tolerant to SO_2_ and sulfite (Noji et al., 2001).

### Remediation of NO_*x*_

There are several oxides of nitrogen (N) in the atmosphere: nitrogen dioxide (NO_2_), nitric oxide (NO), nitrous oxide (N_2_O), nitrogen trioxide (N_2_O_3_), and nitrogen trioxide (N_2_O_5_). Among them, the USEPA regulates NO_2_ only because it is the most prevalent form of NO_*x*_ generated anthropogenically (USEPA, 1999). NO_2_ also participates in the formation of ozone (O_3_) and NO. NO_*x*_ emissions in China increased rapidly from 11.0 Mt in 1995 to 26.1 Mt in 2010. Power plants, industry, and transportation were major sources of NO_*x*_ emissions, accounting for 28.4, 34.0, and 25.4% of the total NO_*x*_ emissions in 2010, respectively (Zhou et al., 2013). The total NO_*x*_ emissions in China are projected to increase 36% based on the 2010 value by 2030.

A group of bacteria like *Azotobacter* and *Rhizobium* and fungi such as mycorrhizas are capable of fixing atmospheric N. *Cyanobacteria* are able of using a variety of inorganic and organic sources of combined N, like nitrate, nitrite, ammonium, urea or some amino acids. These microbes are often associated with plant roots. Nitrifying bacteria including species from the genera *Nitrosomonas, Nitrosococcus, Nitrobacter*, and *Nitrococcus* oxidize ammonia to hydroxylamine, and nitrite oxidoreductase oxidizes nitrite to nitrate. Nitrifying bacteria thrive in soils, lakes, rivers, and streams with high inputs and outputs of sewage, wastewater and freshwater because of high ammonia content. Phyllosphere diazotrophic bacteria, like *Beijerinckia, Azotobacter*, and *Klebsiella* and also Cyanobacteria, such as *Nostoc, Scytonema*, and *Stigonema* can use atmospheric dinitrogen (N_2_) as a source of nitrogen (Whipps et al., 2008). N_2_ is fixed by the nitrogenase enzyme encoded by *nif* genes, and the gene *nifH* has been widely used for analysis of their community structure (Fürnkranz et al., 2008; Rico et al., 2014). The abundance of N_2_-fixing bacteria was also reported to improve drought tolerance, suggesting their adaptability to plants grown in different environmental conditions (Rico et al., 2014).

Plants absorb gaseous NO_2_ more rapidly than NO because NO_2_ reacts rapidly with water while NO is almost insoluble. The uptake of NO_2_ per unit leaf area was reported to be nearly three times that of NO when the two gases occurred in the same concentration (Law and Mansfield, 1982). As a result, NO_2_ has been considered to be more toxic than NO. Visible symptoms resulting from NO_2_ exposure are relatively large, irregular brown or black spots. However, phytotoxicity of NO_2_ is rare and much less than SO_2_ and O_3_. This is due to the fact that NO_*x*_ are plant nutrients. When NO and NO_2_ are absorbed and dissolved in the extracellular solution of leaves, they form nitrate (NO_3_) and NO_2_ in equal amounts and proton (H^+^). NO_3_ is then utilized by plants in the same way as it is absorbed from roots and used as a nitrogen source for synthesizing amino acids and proteins (Figure 1). Foliar absorption of NO_2_ varies widely depending on plant species. Morikawa et al. (1998) studied 217 herbaceous and woody species in uptake of NO_2_ and found that plant species differed by 657 folds in NO_2_ uptake and assimilation. The most efficient woody plants included *Eucalyptus viminalis, Populus nigra, Magnolia kob*u, and *Robinia pseudoacacia*, and the most herbaceous plants include *Erechtites hieracifolia, Crassocephalum crepidioides*, and *Nicotiana tabacum* (Morikawa et al., 1998).

Nitrogen dioxide could be a plant signal molecule that improves plant growth. Morikawa et al. (2004) reported that about one-third of NO_2_-derived N absorbed by leaves was converted into a previously unknown Kjeldahl-unrecoverable organic nitrogen, which comprise a novel heterocyclic Δ2 1,2,3 thiadiazoline derivative and nitroso- and nitro-organic compounds (Miyawaki et al., 2004; Morikawa et al., 2005). These results indicate that NO_2_ is not only known as a pollutant or a supplemental source of N, but also acts as an airborne reactive nitrogen species signal (Morikawa et al., 2004, 2005). This is in agreement with the reports that endogenously produced NO_*x*_ such as NO act as a vital plant signal (Wendehenne et al., 2001; Neill et al., 2003). To further analyze atmospheric NOx effects on plants, Morikawa et al. (2003) determined if plants could use NO_2_ as a fertilizer and concomitantly reduce NO_2_ concentrations. The authors found that application of 282 μg m^−3^ NO_2_, equivalent to the heavily polluted urban air, to plants for 10 weeks almost doubled the biomass, total leaf area, the contents of carbon (C), N, S, phosphorus (P), potassium (K), calcium (Ca), and magnesium (Mg) as well as free amino acid contents and crude proteins (Morikawa et al., 2003). The mass spectrometric analysis of the ^15^N/^14^N ratio showed that N derived from NO_2_ comprised less than 3% of total plant N, meaning that the contribution of NO_2_-N to total N was relatively low. These results imply that NO_2_ could be a multifunctional signal to stimulate plant growth, nutrient uptake, and metabolism (Takahashi et al., 2005).

### Remediation of O_3_

Anthropogenic O_3_ is primarily generated from the reaction of atmospheric O_2_ with ground-state O (3P) radicals that result from the photolytic dissociation of ambient NO_2_. Thus, the presence of NO and NO_2_ in the lower atmosphere is closely linked with ground-level of O_3_. In China, O_3_ levels increased at a rate of 2.2 μg m^−3^ per year from 2001 to 2006. Average O_3_ concentrations in Beijing varied from 45 to 96.2 μg m^−3^ depending on locations (Wan et al., 2014). In Shanghai, 1-h average concentration of O_3_ was 54.2 μg m^−3^. O_3_ level increased during spring, reached the peak in late spring and early summer, and then decreased in autumn and finally dropped in winter. The highest monthly average O_3_ concentration (82.2 μg m^−3^) in June was 2.7 times greater than the lowest level (30.4 μg m^−3^) recorded in December (Zhao et al., 2015).

Ozone is considered an effective antimicrobial agent against some bacteria and fungi (Sharma and Hudson, 2008). There have been no reports on microbial-mediated O_3_ reduction. However, in a study of O_3_ effects on phyllosphere fungal populations, Fenn et al. (1989) found that a chronic exposure of mature Valencia orange trees (*Citrus sinensis*) to O_3_ or SO_2_ for 4 years decreased populations of phyllosphere fungi. In a same experiment conducted by the authors, a short-term fumigation of O_3_ to giant sequoia (*Sequoiadendron giganteum*) and California black oak (*Quercus kelloggii*) did not significantly affect the numbers of phyllospere fungi. Plant absorption of O_3_ is mainly through stomata, O_3_ is easily dissolved in water and reacts with apoplastic structures and plasma membranes to form reactive oxygen species (ROS), such as ${\text{O}}_{2}^{-}$, H_2_O_2_, and OH radical. The O_3_ or ROS can disturb cell membrane integrity and attack sulfhydryl (SH) groups or ring amino acids of protein, thus causing phytotoxicity. Injury symptoms include white, yellow or brown flecks on the upper surface of leaves. The threshold concentrations that cause a 10% reduction in yield are 80μg m^−3^ for sensitive crops and 150 μg m^−3^ for the most resistant crops. Adaptation of plants to O_3_ stress has resulted in plants developing mechanisms against O_3_ toxicity. First, O_3_ can be removed from the air by chemical reactions with reactive compounds emitted by vegetation, particularly monoterpenes (Di Carlo et al., 2004). Second, semi-volatile organic compounds, such as different diterpenoids exuded by trichomes on leaves are an efficient O_3_ sink (Jud et al., 2016). Tobacco leaves can secret diterpenoid cis-abienol, which acts as a powerful chemical protection shield against stomatal O_3_ uptake by depleting O_3_ at the leaf surface. As a result, O_3_ flux through the open stomata is strongly reduced (Jud et al., 2016). As to O_3_ absorbed by leaves, an oxidative burst occurs as the initial reaction to O_3_, followed by activation of several signaling cascade and plant antioxidant systems including ascorbate-glutathione cycle and antioxidant enzymes to alleviate the oxidative burden resulting from O_3_ exposure (Vainonen and Kangasjarvi, 2015).

### Remediation of VOCs

VOCs are organic chemicals that have a low boiling point and a high vapor pressure at room temperature causing large numbers of molecules to evaporate into the surrounding air. VOCs are numerous and ubiquitous including naturally occurring and anthropogenic chemical compounds. VOCs participate in atmospheric photochemical reactions contributing to O_3_ formation and also play a role in formation of secondary organic aerosols, which are found in PMs. The strong odor emitted by many plants consists of green leaf volatiles, a subset of VOCs called biogenic VOCs, which emit exclusively from plant leaves, the stomata in particular. Major species of biogenic VOCs include isoprene, terpenes, and alkanes.

Anthropogenic VOCs include large groups of organic chemicals, such as formaldehyde, polycyclic aromatic hydrocarbons (PAHs), and BTX (benzenes, toluene, and xylenes). The most significant sources of formaldehyde are engineered wood products made of adhesives that contain urea-formaldehyde (UF) resins. BTX come from painting and coating materials used for interior decoration and refurbishment. Motor-vehicle exhausts, tobacco smoke, and heating also contribute to the presence of VOCs. A great concern over VOCs has been indoor air quality. Indoor formaldehyde in recently renovated homes ranged from 0.14 to 0.61 mg m^−3^, and benzene, toluene, and xylenes were 124.0, 258.9, and 189.7 μg m^−3^, respectively (Hao et al., 2014). The formaldehyde concentration is 65–100% higher than indoor air quality standards of China. Formaldehyde and BTX as main indoor VOCs contribute to the so-called “sick building syndrome” (Brown et al., 1994; Wieslander et al., 1996; Wargocki et al., 2000; Berg et al., 2014). This review regarding VOCs is thus emphasized on indoor air quality.

As early as in the 1970s, NASA (U.S. National Aeronautics and Space Administration) conducted research on the use of foliage plants for remediation of air quality in space shuttles. Foliage plants are those with attractive foliage and/or flowers that are able to survive and grow indoors (Chen et al., 2005). Results showed that foliage plants removed nearly 87% of air pollutants from sealed chambers within 24 h (Wolverton et al., 1984, 1989; Cruz et al., 2014a). For example, each plant of peace lily (*Spathiphyllum* spp. ‘Mauna Loa’) removed 16 mg of formaldehyde, 27 mg of trichloroethylen, and 41 mg of benzene from sealed chambers after a 24-h exposure to the respective chemical. Generally, plants absorb gaseous pollutants via leaf stomata. Some of the VOCs are recognized as xenobiotics by plants, and they are detoxified through xenobiotic metabolism, involving oxidoreductase or hydrolases, bioconjugation with sugars, amino acids, organic acids, or peptides, and then removed from the cytoplasm for deposition in vacuoles (Edwards et al., 2011). In addition to plant leaves, rhizosphere microbes also contribute to reduction of VOCs under interior environments (Llewellyn and Dixon, 2011). Using a dynamic chamber technique, Xu et al. (2011) investigated formaldehyde removal by potted foliage plants and found that formaldehyde removal was attributed not only to the formaldehyde dehydrogenase activities of plant leaves but also to the absorption and metabolism by microorganisms in the rhizosphere. Such bacteria have been isolated from soils, water, and different tissues of plants in polluted environments. Many pure cultures of bacteria, including various strains of *P. putida*, have been evaluated for biodegradation of air pollutants. Some fungi strains are also able to use volatile aromatic hydrocarbons as sole source of carbon and catalyze degradation reactions (Prenafeta-Boldú et al., 2001; Kennes and Veiga, 2004; Jin et al., 2006). Here we mainly discuss phylloremediation of formaldehyde, benzene, toluene, and xylene as well as phenols and PAHS.

#### Formaldehyde

Formaldehyde is a colorless, flammable gas or liquid that has pungent and suffocating odor. It poses a significant danger to human health due to its high reactivity with proteins and DNA, thus formaldehyde is known to be a human carcinogen. Plants can directly absorb formaldehyde and transform it to organic acids, sugars or CO_2_ and H_2_O (Figure 1). Giese et al. (1994) exposed shoots of *Chlorophytum comosum* to 8.5 mg m^−3^ gaseous [^14^C]-formaldehyde over 24 h and found that about 88% of the recovered radioactivity was associated with plant metabolites as ^14^C, which had been incorporated into organic acids, amino acids, free sugars, lipids, and cell wall components. Formaldehyde responsive genes were identified from golden pothos (*Epipremnum aureum*) (Tada et al., 2010). Glutathione (GSH)-dependent formaldehyde dehydrogenase (FADH) and formate dehydrogenase (FDH) can detoxify formaldehyde to formate and further to carbon dioxide (Tada and Kidu, 2011). A wide range of foliage plants have been documented to be able to remove formaldehyde. Kim et al. (2010) exposed 86 species of foliage plants individually to 2 μl L^−1^ formaldehyde in sealed chambers and found that formaldehyde removed per cm^2^ leaf area in 5 h ranged from 0.1 to 6.64 mg m^−3^, depending on plant species. The most efficient species in removal of formaldehyde include *Osmunda japonica, Selaginella tamariscina, Davallia mariesii*, and *Polypodium formosanum*. Surprisingly, these efficient plants belong to pteridophytes, commonly known as ferns and fern allies. Why this group of plants is more efficient than the other foliage plants in formaldehyde removal deserves further investigation.

Formaldehyde can also be assimilated as a carbon source by bacteria (Vorholt, 2002). Such assimilation occurs in *Methylobacterium extorquens* through the reactions of the serine cycle (Smejkalova et al., 2010), in *Bacillus methanolicus* through the RuMP cycle (Kato et al., 2006), and in *Pichia pastoris* through the xylulose monophosphate cycle (Lüers et al., 1998). Some fungi also assimilate formaldehyde. Yu et al. (2015) isolated a fungal strain (*Aspergillus sydowii* HUA), which was able to grow in the presence of formaldehyde up to 2,400 mg l^−1^ and the specific activity of formaldehyde dehydrogenase and formate dehydrogenase were as high as 5.02 and 1.06 U mg^−1^, respectively, suggesting that this fungal isolate could have great potential for removing formaldehyde. Some of the bacteria and fungi used to colonize roots can also colonize leaves and could be used for phylloremediation of formaldehyde in the air (Khaksar et al., 2016a).

#### BTX

BTX refers to benzene, toluene, and three xylene isomers [ortho– (or o–), meta– (or m–), and para– (or p–)], which are major components of gasoline. Due to their low water solubility and acute toxicity and genotoxicity, BTX components have been classified as priority pollutants by the USEPA (Eriksson et al., 1998). Plants leaves can absorb BTX mainly through stomata, which are converted to phenol or pyrocatechol, and subsequently to muconic acid and fumaric acid (Ugrekhelidze et al., 1997). Foliage plants, such as *Dracaena deremensis* and *Spathiphyllum* spp. have been documented to remove BTX indoors (Wolverton et al., 1984, 1989; Wood et al., 2006; Mosaddegh et al., 2014). Liu et al. (2007) fumigated 73 plant species with 478.5 μg m^−3^ benzene gas and found that 23 of the 73 species showed inability to reduce fumigated benzene, the rest varied in benzene reduction, ranging from 0.1 to 80%. The most efficient plant species were *Crassula portulacea, Hydrangea macrophylla*, and *Cymbidium* ‘Golden Elf’. Foliage plants that are effective in removal of toluene include *H. helix, Philodendron* spp., *Schefflera elegantisima*, and *Sansevieria* spp. (Kim et al., 2011; Sriprapat et al., 2013; Cruz et al., 2014b). The wax of *Sansevieria trifasciata* and *S. hyacinthoides* is rich in hexadecanoic acid, which could pay an important role in absorption of toluene (Sriprapat et al., 2013). Sriprapat et al. (2014) also evaluated plant absorption of xylene. The tested 15 plant species were able to remove xylene with removal efficiency ranging from 59.1 to 88.2%, of which *Zamioculcas zamiifolia* was the most efficient species.

Bacteria including some strains of *Rhodococcus rhodochrous* (Deeb and Alvarez-Cohen, 1999), *Alcaligenes xylosoxidans* (Yeom and Yoo, 2002), and *P. putida* (Alagappan and Cowan, 2003) and also fungal cultures of *Cladophialophora* sp. (Prenafeta-Boldú et al., 2002) are able to degrade BTX (Figure 1). Many *Pseudomonas* species are leaf colonists and some are plant pathogens (Dulla et al., 2005). BTX are actual growth substrates for a number of organisms, such as *P. putida* (Inoue et al., 1991). In a study of bioremediation of airborne toluene, De Kempeneer et al. (2004) found that the time required for 95% reduction of the initial toluene concentration of 339 mg m^−3^ was 75 h by *Azalea indica* plants along. Such reduction by the plants inoculated with *P. putida* TVA8 under the identical conditions was only 27 h. Subsequent additions of toluene further increased the removal efficiency of plants inoculated with the bacterial strain, but the toluene-removal rate was comparably low in plants without inoculation. Hence, inoculation of the leaf surface with *P. putida* TVA8 was considered to be essential for rapid removal of toluene. These results clearly demonstrated the importance of both plant leaves and leaf-associated microbes in phylloremediation of indoor air pollutants. The genetics and biochemistry of strains F1 and mt-2 of *P. putida* have been intensively studied (Harayama and Rekik, 1990; Horn et al., 1991; Timmis et al., 1994; Aemprapa and Williams, 1998). Such information could be important for exploring these strains for effective removal of air pollutants.

#### Air borne phenols and polycyclic aromatic hydrocarbon (PAHs)

Air borne phenols are a class of chemical compounds containing a hydroxyl group bonded directly to an aromatic hydrocarbon group, whereas PAHs are hydrocarbon comprising only carbon and hydrogen with multiple aromatic rings. Phenol and PAHs are major air pollutants in urban areas, and some PAHs have been considered carcinogenic. It has been reported that *Bacillus cereus* can degrade phenol via meta-cleavage pathway (Banerjee and Ghoshal, 2010). *Pseudomonas* sp. CF600 can mineralize phenol on bean and maize leaves by dmp catabolic pathway (Sandhu et al., 2007). Sandhu et al. (2007) directly measured phenol degradation by natural phyllosphere communities. Leaves were collected from trees growing in an area that was known to have high concentrations of VOCs. Unsterilized and surface-sterilized leaves were then exposed to radiolabeled phenol in closed chambers for 24 h and the amount of phenol degradation was compared. The phenol degradation by the non-sterilized leaves was significantly greater than the degradation by the sterilized leaves, indicating that degradation of VOCs was enhanced by the presence of the phyllosphere communities. This work indicates that plant leaves can accumulate phenols, which may be subsequently available for bacteria in the phyllosphere for degradation.

Plant leaves can absorb atmospheric PAHs. A study on deciduous forest in Southern Ontario, Canada, confirmed that amounts of phenanthrene, anthracene, and pyrene were reduced within and above the forest canopy during bud break in early spring (Choi et al., 2008). Plant species differ in removal of PAHs, the differences could be attributed to specific morphological and chemical constitutions of plants as well as leaf-associated microbes. Phyllosphere bacteria on 10 ornamental plant species were studied based on their diversity and activity toward the removal of PAHs (Yutthammo et al., 2010). The phyllosphere hosted diverse bacterial species including *Acinetobacter, Pseudomonas, Pseudoxanthomonas, Mycobacterium*, and unculturable ones, of which PAH degrading bacteria accounted for about 1–10% of the total heterotrophic phyllosphere populations depending on plant species. The analysis of bacterial community structures using PCR and denaturing gradient gel electrophoresis showed that each plant species had distinct band patterns, suggesting that the bacterial communities are closely associated with leaf morphology and chemical characteristics of ornamental plant species. Furthermore, branches of fresh leaves of selected plant species were evaluated in sealed chambers for removal of a mixture of PAHs (acenaphthene, acenaphthylene, fluorene, and phenanthrene). Bacteria on unsterilized leaves of all tested plants showed an enhanced removal of phenanthrene. Bacteria on leaves of *Wrightia religiosa* in particular were able to reduce all the tested PAHs (Yutthammo et al., 2010). Therefore, phyllosphere bacteria on ornamental plants may play an important role in natural attenuation of airborne PAHs and plant species differ in supporting microbes in PAH removal.

## Development of phylloremediation technologies

**Figure 2:**
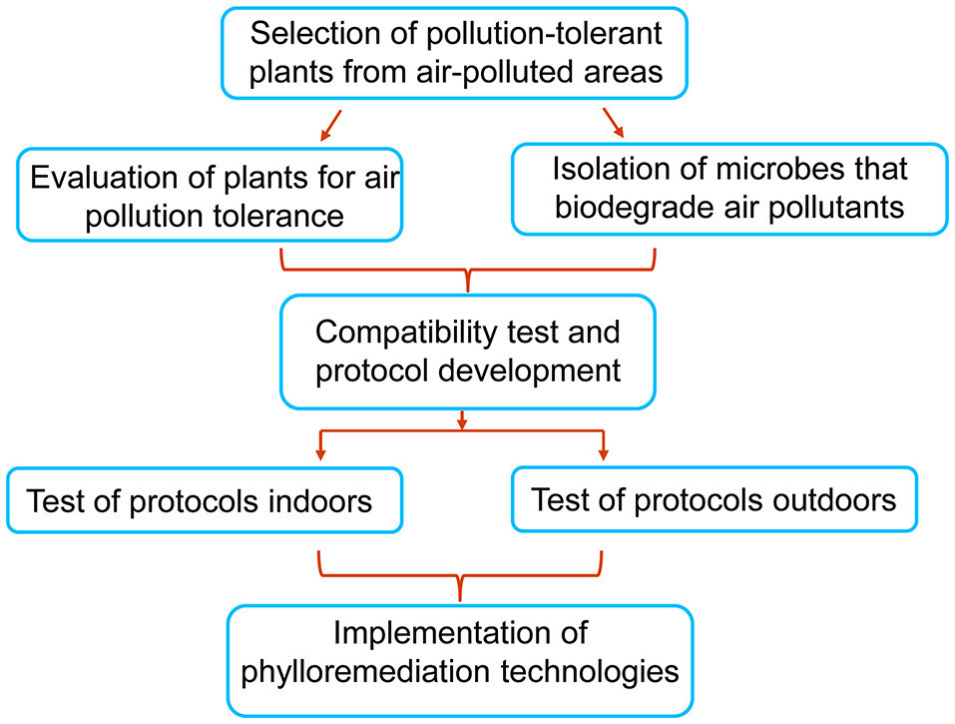
A general outline for developing phylloremediation technologies. Plants species and microbes should be selected from air polluted areas. Selected plants should be evaluated for their ability to adsorb or absorb air pollutants, and concurrently microbes are screened for biodegradation or biotransformation of pollutants. The selected plants and microbes are tested for synergistic effects on the reduction of particular air pollutants. Based on the test results, specific plant-microbe combinations that can remove one or more air pollutants are identified, and protocols are formulated for evaluating their effectiveness in removal pollutants indoors and outdoors. Effective protocols will be developed into phylloremediation technologies for use in reducing air pollutants.

This review has documented that plant leaves and leaf-associated microbes individually can reduce air pollution and the combination of the two generally exhibits enhanced remediation of air pollutants. Since air pollution never before has become such an urgent problem in countries like China and India, now is the time to seriously consider all options for reducing the pollutants. Phylloremediation is a natural and environmentally friendly way of bioremediation of air contaminants. Our proposal for developing phylloremediation technologies is outlined in Figure 2, which includes (1) selection and evaluation of appropriate plant species and microorganisms that are tolerant to pollution and able to remove one or more air pollutants; (2) testing and analysis of the compatibility of plant leaf surfaces with isolated microbes for synergetic interactions in reduction of pollutants in laboratories, in simulated indoor environments, and in outdoor settings; (3) analysis of experimental data and development of phylloremediation technologies; and (4) implementation of the technologies for remediation of air in both indoor and outdoor environments.

### Plant selection

Plants should be selected from four categories: (1) trees, (2) shrubs or small tress, and (3) ground cover plants for use in outdoor environments as well as (4) foliage plants for indoor environments. Trees are referred to as perennial plants with elongated stems or trunks, supporting branches and leaves. Shrubs (or small trees) are those small to medium-sized woody plants that grow under some degree of shaded conditions. Ground covers are any plants that can grow over an area of ground and they can grow below the shrub layer including turfgrass and other woody and herbaceous selections. Foliage plants are those which can grow and survive indoors for interior decoration.

Plant species not only differ greatly in adsorption, absorption, and assimilation of air pollutants but also vary significantly in pollution tolerance. Air pollution tolerance index has been used for evaluation of plants specie in response of pollutants (Singh et al., 1991). Information generated by the index is useful, but the index may require revision for better reflecting the ability of plants in tolerance of air pollutants. An initial large-scale evaluation of plants from the four categories should be conducted for identifying candidate species that are able to tolerate PMs, O_2_, SO_2_, NO_*x*_, and VOCs individually or collectively and can also substantially retain or assimilate these pollutants. Plants should also tolerate abiotic stresses, such as drought, heat, and cold, and biotic stresses like plant pathogens. Leaves of plants should be able to support one or more selected microbes. Trees should have a relatively fast growth rate. Needle-leaved plants should be particularly considered. As mentioned before, needles are rich in waxes for capturing PMs, and they are also used as as passive bio-samplers to determine polybrominated diphenyl ethers (Ratola et al., 2011). Broad-leaved plants should have more hairs or trichomes and more stomata with a large canopy. Leaf water and nutritional contents, leaf cuticular wax composition, hairs or trichomes, and surface physical characteristics should be suitable for microbial colonization. Shrubs and ground cover plants should have similar leaf physical and chemical properties but be able to tolerate slight shade. For foliage plants, they should substantially tolerate shade and can survive and grow under indoor low-light conditions.

Plant species possessing the aforementioned traits should be selected from particular regions where plants survive and thrive under heavily polluted environments. The rationale is that plants that are able to grow in the polluted environments may develop mechanisms for adaptation to the stressful conditions. Thus, some regions of China and India could be ideal locations for initial selection of plant species. Plants have been documented to tolerate multiple stresses, which include induced cross tolerances and the ability of particular variants to resist multiple distinct stresses. Reactive oxygen species are key molecular signals produced in response to multiple stresses, which are aimed at the maintenance of cellular equilibrium (Perez and Brown, 2014). Glutathione-S-transferase (GST) genes play an important role in the maintenance of ROS equilibrium. Salicylic acid, jasmonic acid, and ROS interplay in the transcriptional control of multiple stresses. Additionally, omics technologies should be used for identifying molecular mechanisms in regulation of plant responses to multiple stresses. Such information, particularly transcriptional factors, key regulatory genes or enzymes should be incorporated into the plant selection processes.

Genetic engineering is an option for improving plants to remediate air pollutants (Abhilash et al., 2009). Genes listed in Table 4 can be used for generating transgenic plants. Cysteine synthase is a key enzyme to utilize H_2_S and SO_2_ as a sulfur source to synthesize cysteine. Overexpression of cysteine synthase in rice was shown to enhance sulfur assimilation upon exposure to a high level of H_2_S (Yamaguchi et al., 2006). Nitrite reductase catalyzes the six-electron reduction of nitrite to ammonium. Transgenic *Arabidopsis* plants bearing chimeric spinach *NiR* gene enhanced nitrite reductase activity and NO_2_ assimilation (Takahashi and Morikawa, 2001). Cytochrome P450 2E1 has strong and specific capacity of decomposing organic pollutants in animal bodies. Transgenic tobacco plants overexpressing *CYP2E1* gene showed increased ability to detoxify broad classes of pollutants such as chlorinated solvents and aromatic hydrocarbons (James et al., 2008). Unlike tobacco, poplar (*Populus tremula* × *Populus alba*) plants are a fast-growing tree species with large canopies. Poplar plants overexpressing a mammal *CYP2E1* exhibited increased metabolism and enhanced removal of organic pollutants from hydroponic solution and the air (Doty et al., 2007). Some genes from microbes can also be used for engineering transgenic plants for phylloremediation. The ribulose monophosphate (RuMP) pathway is one of the formaldehyde-fixation pathways found in microorganisms (Orita et al., 2006). The key enzymes of this pathway are 3-hexulose-6-phosphate synthase (HPS), which fixes formaldehyde to D-ribulose 5-phosphate (Ru5P) to produce D-arabino-3-hexulose 6-phosphate (Hu6P) and 6-phospho-3-hexuloisomerase (PHI), and then converts Hu6P to fructose 6-phosphate (F6P) (Orita et al., 2006; Chen et al., 2010). Co-expression of HPS and PHI in tobacco plants resulted in 20% reduction of formaldehyde compared to the control plants (Chen et al., 2010). In another study, a chlorocatechol 1,2-dioxygenase gene (*tfdC*) derived from the bacteria *Plesiomonas* was introduced into *Arabidopsis thaliana* (Liao et al., 2006). Transgenic plants showed enhanced tolerances to catechol, an aromatic ring. Transgenic plants were also able to remove a large amount of catechol from their media and highly efficient in convertion of catechol to cis, cis-muconic acid, suggesting that degradative genes derived from microbes can be used to produce transgenic plants for bioremediation of aromatic pollutants in the environment (Liao et al., 2006).

**Table 4:** Genes from different sources have been demonstrated to be able to remediate air pollutants in transgenic plants.

**Gene name**	**Source**	**Transgenic plants**	**Pollutant**	**References**
3-hexulose 6-phosphate and 6-phospho-3-hexuloisomerase	*Mycobacterium gastri* MB19	*Arabidopsis thaliana* and *Nicotiana tabacum*	Formaldehyde	Chen et al., 2010
Biphenyl-Chlorobiphenyl Dioxygenase	*Burkholderia xenovorans*	*Nicotiana tabacum*	4-chlorobiphenyl	Mohammadi et al., 2007
Chlorocatechol 1,2-dioxygenase	*Plesiomonas* spp.	*Arabidopsis thaliana*	Catechol	Liao et al., 2006
CYP450 2E1	*Oryctolagus cuniculus*	*Populus tremula* × *populous alba*	Trichloroethylene, benzene, and chloroform	Doty et al., 2007
CYP450 2E1	*Homo sapiens*	*Nicotiana tabacum*	Trichloroethylene, and ethylene dibromide	Doty et al., 2000
CYP450 2E1	*Homo sapiens*	*Nicotiana tabacum*	Trichloroethylene, benzene, toluene, vinyl chloride, chlorotoluene, and chloroform	James et al., 2008
Cysteine synthase	*Nicotiana tabacum*	*Nicotiana tabacum*	SO_2_	Noji et al., 2001
Glutathione reductase	*Escherichia coli*	*Populus sieboldii* × *P. grandidentata*	SO_2_	Endo et al., 1997
Mn-peroxidase	*Coriolus versicolor*	*Nicotiana tabacum*	Phencyclidine	Iimura et al., 2002
Nitrite reductase	*Spinacia oleracea*	*Arabidopsis thaliana*	NO_2_	Takahashi and Morikawa, 2001
O-acetylserine(thiol) lyase	*Triticum aestivum*	*Nicotiana tabacum*	Hydrogen sulfide	Youssefian et al., 1993
O-acetylserine(thiol) lyase	*Triticum aestivum*	*Nicotiana tabacum*	SO_2_	Youssefian et al., 2001
Peroxidases	*Lycopersicon esculentum*	*Nicotiana tabacum*.	Phenol	Sosa Alderete et al., 2009

Selected plants should be evaluated in controlled environmental chambers to measure their capacity for tolerance and also assimilation of air pollutants. Seedlings could be exposed to particular pollutants or a mixture of pollutants in different concentrations and durations. Plant responses to the exposures could quickly evaluated based on stomatal conductance, net photosynthetic rate, the maximum quantum efficiency of photosystem II using the new LI-COR6800. Their morphological appearance, i.e., leaf greenness, leaf size, and plant height and canopy dimension compared to control treatments should be evaluated. The ability of plants to remove pollutants should be tested using GC-MS. For evaluation of plant responses to PM, in addition to the mentioned plant characteristics, leaf morphology, particularly leaf surface characters should be examined under microscopes and stomatal size and density recorded. If needed, isotopic labeling techniques could be used to track the fate of particular compounds. The evaluation results once analyzed and compared, plants that tolerate stresses and are able to adsorb or absorb or assimilate pollutants could be identified from each type of plants for subsequent compatiablity tests with selected microbes.

### Microbe selection

Cultivable bacteria only account for a small fraction of the total diversity in the phyllosphere, which has greatly hampered the use of some valuable microbes. New approaches, such as the use of improved culture and advanced devices (i-Chip), co-culture with other bacteria, recreating the environment in the laboratory, and combining these approaches with microcultivation should be employed to convert more uncultivable bacteria into cultured isolates in the laboratory (Nichols et al., 2010; Stewart, 2012; Müller and Ruppel, 2014). Similar to plant selection, initial microbial selection could be carried out in areas where plants have been contaminated by air pollutants. In coordination with plant selection, microbes could be isolated from leaves of plants identified in plant selection. This is because the pollutants may exert selective pressures to phyllosphere microbial diversity. For example, bacterial communities hosted by *Platanus* × *acerifolia* leaves from different locations of Milan (Italy) were analyzed by high throughput sequencing. The results showed that biodiversity of bacterial communities decreased but hydrocarbon-degrading populations increased along the growing season, which suggest that air contaminants might play an important role in the selection of phyllospheric populations in urban areas (Gandolfi et al., 2017).

A particular attention should be given to endophytic microbes. There are about 300,000 plant species on the earth; each plant could host one or more endophytes (Petrini, 1991; Strobel and Daisy, 2003). Endophytes are resided inside plant tissues and generally have no harmful effects on plants. Endophytic bacteria that colonize leaves could be particularly desirable as they could not be washed away by precipitation. Recent advances in endophyte-assisted remediation have been reviewed (Khan and Doty, 2011; Stepniewska and Kuzniar, 2013; Ijaz et al., 2016; Syranidou et al., 2016). Endophytic *B. cereus* ZQN5 isolated from natural *Zamioculcas zamiifolia* leaves enhanced ethylbenzene removal rate on sterile *Z. zamiifolia* (Toabaita et al., 2016). Microbes could also be isolated from the rhizosphere of plants contaminated by air pollutants as more endophytism occurs in roots (Ijaz et al., 2016). Some of leaf endophytes could be initially established in roots and subsequently transported to shoots. Khaksar et al. (2016a) reported that some microbes isolated from roots can also colonize leaf surfaces. An endophytic strain of *B. cereus* ERBP from roots of *Clitoria ternatea* was able to colonize the leaf surface of *Z. zamifolia*. During a 20-d fumigation with formaldehyde, the inoculation of ERBP did not interfere with the natural shoot endophytic community of *Z. zamiifolia*. ERBP inoculated *Z. zamiifolia* exhibited a significantly higher formaldehyde removal efficiency when compared to the non-inoculated plants.

Microbes, once identified and cultured, could be engineered to improve phylloremediation capacity (Table 5). A pTOM toluene-degradation plasmid from *B. cepacia* G4 was introduced into *Bacillus cepacia* L.S.2.4, a natural endophyte from yellow lupine (*Lupinus arboreus*; Barac et al., 2004). After the engineered bacteria were inoculated into aseptic lupine seedlings, the recombinant endophytics degraded 50–70% more toluene and provided much more protection against the phytotoxic effects of toluene than that obtained from soil bacteria (Barac et al., 2004). Horizontal genes can transfer among plant-associated endophytic bacteria in plants. Poplar was inoculated with the yellow lupine endophyte *B. cepacia* VM1468, which contains the pTOM-Bu61 plasmid coding for constitutively expressed toluene degradation (Taghavi et al., 2005). Inoculated plant growth was enhanced in the presence of toluene, and the amount of toluene release via evapotranspiration was also reduced. Although no inoculated strains were detected in the endophytic community, there was horizontal gene transfer of pTOM-Bu61 to different members of the endogenous endophytic community (Taghavi et al., 2005). The TCE-degrading strain *P. putida* W619-TCE also can be engineered via horizontal gene transfer in poplar plants (Weyens et al., 2009b).

**Table 5:** Genes from microbes have been demonstrated to be able to remediate pollutants in transgenic microbes.

**Gene name**	**Source**	**Transgenic organism**	**Pollutant**	**References**
BphA1	*Burkholderia xenovorans* LB400	*Pseudomonas pseudoalcaligenes*	Aromatic hydrocarbons and pentachlorobenzene	Suenaga et al., 2010
C23O	*Pseudomonas aeruginosa* zl1f4	*Bacillus subtilis*	Phenol	Yang et al., 2012
Camphor monooxygenase and a hybrid dioxygenase	*Pseudomonas putida*	*Alcaligenes*	Pentachloroaniline	Iwakiri et al., 2004
Catechol 2, 3-dioxygenase	*Pseudomonas aeruginosa* SZH16	*Pseudomonas fluorescens* P13	Phenol	Yang et al., 2012
Hemoglobin	*Vitreoscilla* sp.	*Pseudomonas putida*	Benzene, toluene and xylene	Kahraman and Geckil, 2005
Hemoglobin	*Vitreoscilla* sp.	*Xanthomonas maltophilia*	Benzoic acid	Liu et al., 1996
Phenol hydroxylase	*Escherichia coli*	*Pseudomonas putida*	Trichloroethylene	Fujita et al., 1995
Phenol Hydroxylase	*Ralstonia* sp.	*Ralstonia* sp.	Trichloroethylene	Ishida and Nakamura, 2000
Pro U operon	*Escherichia coli*	Microbial consortium	Hydrocarbon	Kapley et al., 1999
Tod and xyl	*Pseudomonas putida*	*Deinococcus radiodurans*	Toluene	Brim et al., 2006
Toluene dioxygenase	*Deinococcus radiodurans*	*Deinococcus radiodurans*	Toluene and Trichloroethylene	Lange et al., 1998
Toluene o-monooxygenase	*Burkholderia cepacia*	*Pseudomonas fluorescens*	Trichloroethylene	Yee et al., 1998
Xyl and lux gene cassette	*Pseudomonas putida*	*Pseudomonas putida*	Xylene	Kong et al., 2005

Efforts on microbe selection should also be placed on the identification of microbes that could remediate PM, SO_2_, NO_2_, and O_3_. As mentioned above, a group of microbes can assimilate SO_2_ and NO_2_, further research should explore those microbes for effective assimilation of the two pollutants. Thus far, it appears that no information is available regarding microbial remediation of PM and O_3_, which may not be the case in the nature. Extensive research should be conducted to determine if nature has offered microbes that can break down PMs and can also biodegrade or biotransform O_3_.

Selected microbes could be domesticated by growing them in different cultures varying in pH, carbon source, temperature, and O_2_ to identify appropriate culture media and conditions for maximizing their growth. Morphological characterization and internal transcribed spacer rDNA analysis should be conducted to determine their phylogenetic relationships with other microbes. Their ability to biodegrade particular or a group of air pollutants should be evaluated in the laboratory. Microbial characteristics including their utilization of organic compounds, decomposition rate of pollutants, adaptability, competition, and growth rate should be recorded and analyzed. Competitive strains that show promise in bioremediation should be identified. A series of bacterial and filamentous fungal genomes have been sequenced recently. More than hundreds of bacterial and fungal transcriptomic and proteomic datasets are available. With the advent of increasingly sophisticated bioinformatics and genetic manipulation tools, mechanisms underlying the biodegradation or transformation of pollutants by the isolated microbes could be elucidated. This information, in turn, will significantly improve our understanding of the microbes and provide us with molecular bases for manipulation of the microbes for enhancing phylloremediation.

### Evaluation of the compatibility between plant leaves and microbes

Plants selected from the four categories should be inoculated with selected microbes to determine the compatibility of each selected microbe with each selected plant species. The test could begin first in laboratory settings using entire leaves in designated chambers or utilizing young seedlings in relative large growth chambers to evaluate if inoculated microbes could grow on leaf surfaces and if the specific inoculation affects plant growth. Compatible combinations would be exposed to pollutants at different concentrations and durations to determine the potential for pollutant reduction. A microbe that is compatible with one plant species may not be compatible with another. For example, *B. cereus* ERBP isolated from roots of *C. ternatea* was compatible with the leaf surface of *Z. zamifolia* but not with the leaf surface of *Euphorbia milii*. ERBP-colonized *Z. zamifolia* grew well and showed high efficiency in removal of formaldehyde, but ERBP-colonized *E. milii* were less effective in removal formaldehyde and the plants exhibited stress symptom (Khaksar et al., 2016a). Laboratory evaluation will generate a large number of plant-microbe combinations that are specifically effective in removal of a particular pollutant or a particular group of pollutants. Bacteria would be propagated using bioreactors and corresponding plants would be propagated through either cuttings or tissue culture. The plants would be transplanted into greenhouses or specific regions with air pollution for testing the effectiveness of the combinations in real-world situations.

Plants and microbe combinations that pass the real-world test will be investigated using the next-generation sequencing (NGS) technologies (metagenomics, metatranscriptomics, metaproteomics, and metabolomics) and the rapid evolution of SIP (Stable isotope probing) for identifying molecular mechanisms underlying microbial and plant interactions in facilitation of phylloremediation. The compatibility evaluation and molecular analysis would ultimately result in the development of protocols for culturing microbes and producing corresponding plants. Some protocols will be catered to trees, others used for shrubs or small trees. Some would be effective for improving groundcover plants, and some will be used for indoor foliage plants. Effectiveness of each protocol in remediation of particular or general pollutants would be determined using the model described by Nowak et al. (2006). If the test is to be conducted in a large scale, satellite image acquisition and analysis should be used. The analysis of the data will finally validate the protocols, i.e., particular plants can be inoculated with a specific group of microbes for use in remediation of a particular pollutant or a mixture of pollutants.

### Implementation of phylloremediation technologies

The protocols will be implemented for phylloremediation. We propose three types of plantscape: (1) manufactory plantscape, (2) urban plantscape, and (3) interior plantscape. The plantscape for manufactories and cities should have three levels of greening: the sky with trees, the ground with groundcover plants, and shrubs in between. Additionally, climber plants can be used to build green walls and small trees and shrubs as well as groundcovers can be used to build green roofs. For interior plantscape, each room should have a minimum of one potted foliage plant. Foliage plants can also be used to install green walls in interior environments for enhance remediation of indoor air pollutants.

The implementation of phylloremediation technologies should also take landscape design concepts into consideration, resulting greenbelts, green parks, green walls that fulfill roles not only for air remediation but also for recreation. Depending on the occurrence of pollutants and the scale and degree of the overall pollution, relevant protocols to the particular situations would be implemented. The remediation efficiency could be monitored over time using specific models in connection with satellite imagine data to determine how much of individual pollutants have been removed.

## Conclusion

Air pollution is real, and it is adversely affecting human comfort and health and jeopardizing the ecosystem. The causes are multidimensional including increased population, urbanization, and industrialization accompanied with increased energy consumption and economic growth along with weak regulation, deforestation, and climate change. A recent article published by Cai et al. (2017) suggested that circulation changes including the weakening of the East Asia winter monsoon induced by global greenhouse gas emission contribute to the increased frequency and persistence of the haze weather conditions in Beijing, China. This claim could be true. The fact is that air pollutants released anthropogenically has caused the global warming. Our attention nevertheless should focus on how to control the emissions and how to remediate the pollutants. Although rhizosphere (roots and root associated microbes) contributes greatly to remediation of air pollutants, in this review, we specifically discuss phylloremediation. The role of plant leaves and leaf-associated microbes in remediation of air pollutants has not been well explored. Using the Urban Forest Effects Model, Yang et al. (2005) studied the influence of the urban forest on air quality in Beijing, China and found that the 2.4 million trees in the central part of Beijing removed 1,261.4 tons of pollutants from the air in 2002, of which 720 tons were PM. Nowak et al. (2014) has shown that computer simulations with local environmental data reveal that trees and forests in the contiguous US removed 17.4 million tons (t) of air pollution in 2010, with human health effects valued at 6.8 billion US dollars. Such forest-aided remediation might have avoided more than 850 incidences of human mortality and 670,000 incidences of acute respiratory problems.

We believe that phylloremediation is an environmentally friendly, cost effective way of remediation of air pollutants. The key component of this technology lies in plants. It is plants that can adsorb or absorb pollutants and plants that support microbes in biodegradation or biotransformation of pollutants. To develop phylloremediation technologies, some basic questions should be addressed: (1) Anatomical, physiological, biochemical and molecular mechanisms underlying plant responses to each pollutant should be investigated. Previous research has documented plant responses to pollutants such as NO_*x*_, SO_2_, O_3_, and VOCs, but the research was largely intended to identify how plants were injured. We need to exploit why many plants are tolerant to the pollutants, what are the underling mechanisms, and how can we manipulate the mechanisms for increased tolerance and for use in phylloremediation. There is little information regarding plant responses to PM. Do plants simply adsorb PM? What are the fates of stomatal absorbed PM? (2) Phyllosphere microbes are still largely a mystery and many are not culturable. Methods for collection, identification, and cultivation should be developed. Some microbes isolated from the rhizosphere can also be used for leaf colonization. Mechanisms for biodegradation and transformation of pollutants have been mentioned in this review. However, we still do not know if there are microbes that can remediate PM and O_3_. An important question that should be immediately addressed is the roles of microbes within the PM. Do the microbes become active once settled on leaves? Do they have the ability to break down the PM? With the advances of omics, these questions will be answered, and new strains with high efficiency in breaking down pollutants are expected to be isolated and utilized. (3) A large scale and intensive test for the compatibility among identified plants and identified microbes should be carried out. Specific plant-microbe groups or combinations that can effectively reduce one or more pollutants should be identified, tested, and confirmed in real-world situations and corresponding protocols for using each combination should developed. (4) New methods for analyzing dynamic changes of air pollutants in the atmosphere should be developed and standardized for monitoring the effectiveness of the phyllosphere technologies. (5) Research and development of phyllosphere technologies is a multidisciplinary project requiring collaboration among researchers with different academic backgrounds at regional, national, and international levels. Nature has offered healthy alternatives for remediation of air pollution; we should collaborate with nature as a partner to restore nature's identity.

## Author contributions

All authors contributed to the acquisition and interpretation of available literature and the conception of the work. JC, SL, and XW wrote the manuscript, and all authors reviewed and revised the manuscript and approved this final version. XW and SL contributed equally to this work.

### Conflict of interest statement

The authors declare that the research was conducted in the absence of any commercial or financial relationships that could be construed as a potential conflict of interest.
